# A First-in-Human Study of ATM Inhibitor Lartesertib as Monotherapy in Patients with Advanced Solid Tumors

**DOI:** 10.1158/1078-0432.CCR-25-1648

**Published:** 2025-08-28

**Authors:** Lillian L. Siu, Timothy A. Yap, Sofia Genta, Gregory Pennock, Christine Hicking, Deepthi S. Vagge, Jatinder Kaur Mukker, Giuseppe Locatelli, Anthony W. Tolcher

**Affiliations:** 1 Princess Margaret Cancer Centre, University Health Network, Toronto, Canada.; 2 University of Texas MD Anderson Cancer Center, Houston, Texas.; 3 Kingston Health Sciences Centre, Kingston, Canada.; 4 EMD Serono, Billerica, Massachusetts.; 5 The Healthcare Business of Merck KGaA, Darmstadt, Germany.; 6 Merck Specialities Pvt. Ltd., Bengaluru, India, an affiliate of Merck KGaA, Darmstadt, Germany.; 7 New Experimental Therapeutics (NEXT), San Antonio, Texas.

## Abstract

**Purpose:**

This first-in-human phase I, open-label study (NCT04882917) evaluated the safety, tolerability, pharmacokinetics (PK), pharmacodynamics (PD), and maximum tolerated dose (MTD) of the highly potent and selective oral ataxia-telangiectasia–mutated kinase inhibitor lartesertib.

**Patients and Methods:**

Patients with advanced solid tumors received oral doses of lartesertib for a dose range of 100 to 400 mg once daily. Dose escalation was based on PK, PD, and safety data guided by a Bayesian two-parameter logistic regression model. Molecular responses were assessed in ctDNA samples.

**Results:**

Twenty-two patients received lartesertib at doses of 100 mg (*n* = 2), 200 mg (*n* = 7), 300 mg (*n* = 9), and 400 mg (*n* = 4) once daily. Maculopapular rash was the most common dose-limiting toxicity (four events in four patients). The MTD was 300 mg once daily. The most common grade ≥3 treatment-emergent adverse event was anemia (four patients). Five patients experienced ≥1 treatment-related adverse events of grade ≥3 (including one grade 4 event of hypersensitivity). Exposure increased in a dose-related manner, with median time to maximum plasma concentration ranging from 1 to 2 hours and mean elimination half-life from 5 to 7 hours across the dose range. PD analysis showed a trend of reduction of γ-H2AX levels, with highest target inhibition of 80% to 100%. Best overall response was stable disease in two patients. Molecular responses were observed in four patients of 21 evaluable patients.

**Conclusions:**

Lartesertib achieved target exposure and engagement without significant hematological toxicity. Further clinical evaluation of lartesertib in combination therapy is ongoing.

**Translational Relevance**

This first-in-human phase I study demonstrates that the ataxia-telangiectasia–mutated kinase inhibitor lartesertib is well tolerated and exhibits a manageable safety profile in adult patients with advanced solid tumors. The maximum tolerated dose was established at 300 mg once daily. Lartesertib exposure increased in a dose-related manner, with minimum accumulation after multiple doses. Pharmacodynamic analyses indicated a trend of reduction in γ-H2AX levels, with highest target inhibition of 80% to 100%. No significant effects of lartesertib monotherapy on the immunophenotype, molecular responses, or circulatory factors were detected. Based on the potential of lartesertib as a combination partner with other DNA damage response inhibitors, ongoing clinical trials are evaluating lartesertib in combination with the ATR inhibitor tuvusertib in patients with advanced solid tumors and epithelial ovarian cancer.

## Introduction

The DNA damage response (DDR) is an integrated cellular surveillance and signaling network activated by endogenous/exogenous DNA damage and plays a key role in preventing genomic instability, a hallmark of cancer (1–3). Ataxia-telangiectasia mutated (ATM), a serine/threonine kinase of the PI-3 kinase–like protein kinase family, is a master regulator of the DDR. Upon detecting double-strand breaks (DSB), ATM activates multiple downstream effectors, including checkpoint kinase 2 and p53, and promotes DSB repair through homologous recombination (4, 5).

ATM contributes to the development of resistance to antitumor agents by repairing DNA damage, including DSB caused by chemotherapy and radiotherapy, allowing affected cells to survive (6, 7). Hence, ATM is an attractive target to inhibit the repair of therapy-induced DSB and improve the efficacy of DNA damage–inducing anticancer therapies (8–11). ATM signaling is upregulated in several cancers (i.e., breast, prostate, pancreatic, and melanoma; refs. 5, 12), supporting the rationale for the potential use of ATM inhibition in anticancer therapy. Moreover, ATM inhibition may represent an effective strategy in cancers with impairment of other DDR genes due to synthetic lethality, a phenomenon known to significantly impact sensitivity to other DDR-directed cancer drugs, such as PARP inhibitors (13, 14).

Lartesertib is a potent and selective, orally administered inhibitor of ATM that has been shown to potentiate the effects of DNA DSB-inducing agents (15–17). *In vitro,* lartesertib enhances the radiotherapy sensitivity of diverse cancer cell lines (head and neck, colon, lung, gastric, breast, and melanoma) in a dose-dependent manner (16). In a human squamous cell carcinoma of the head and neck FaDu xenograft model, lartesertib has demonstrated significant dose-dependent antitumor responses in combination with radiation (16). The combination of lartesertib with the ATR inhibitor gartisertib resulted in complete tumor growth inhibition in the MiaPaCa2 pancreatic cancer model and led to almost complete tumor regression in the acute myeloid leukemia model Mv4.11 (17). Furthermore, combining lartesertib with PARP inhibitors (rucaparib or niraparib) demonstrated enhanced efficacy and acceptable tolerability in an HBC-x9 triple-negative breast cancer model that expresses wild-type *BRCA1/2*. These findings further support the rationale for clinical investigation of lartesertib alone or in combination with another DDR inhibitors in patients with advanced tumors (16).

Herein, we report the results of the DDRiver Solid Tumors 410 study part 1A, a phase I, first-in-human study evaluating the safety and tolerability of lartesertib as monotherapy in patients with advanced solid cancers (NCT04882917).

## Patients and Methods

### Study design and treatment

DDRiver Solid Tumors 410 was an open-label, first-in-human, multicenter, phase I study of lartesertib monotherapy, continuously administered once daily, conducted at three study sites in the United States and Canada. Part 1A was a lartesertib monotherapy dose-escalation study designed to evaluate the safety, tolerability, pharmacokinetics (PK), pharmacodynamics (PD), and early signs of efficacy in patients with advanced solid tumors. Patients were treated with lartesertib at increasing dose levels (DLs) ranging from 100 to 400 mg once daily. Each DL included a dose-limiting toxicity (DLT) observation period of 21 days; treatment was continued until disease progression, death, consent withdrawal, unacceptable toxicity, or the end of the study. Additional details are provided under Study Design in the Supplementary Material S1.

The study was approved by the Institutional Review Board or Independent Ethics Committee of each center and was conducted in accordance with the Declaration of Helsinki, the International Conference on Harmonisation Good Clinical Practice guidelines, local laws, and applicable regulatory requirements. The study protocol and other relevant documents were reviewed and approved by an Institutional Review Board/Independent Ethics Committee before study start, and all patients provided their written informed consent for participation.

### Patients

Eligible patients were ≥18 years of age with advanced solid tumors for whom no standard-of-care therapy existed or was not considered sufficiently effective or who could not tolerate standard-of-care. Additional eligibility criteria included an Eastern Cooperative Oncology Group performance status of ≤1 and adequate hematologic, hepatic, and renal function. Patients with clinically significant uncontrolled intercurrent illness and patients with tumors harboring previously identified ATM mutations were excluded (full inclusion and exclusion criteria are provided in the Supplementary Material S1). The representativeness of the study participants is described in Supplementary Table S1.

### Study endpoints and assessments

The primary endpoints were the occurrence of DLTs, adverse events (AE), and treatment-related and treatment-emergent AEs (TEAE), along with the occurrence of clinically significant changes in vital signs, laboratory parameters, and 12-lead electrocardiogram findings. The secondary endpoints included PK parameters of lartesertib in plasma after single and multiple doses; establishment of the recommended dose for expansion based on safety, PK, and PD data; absolute and relative changes overtime from baseline in ATM pathway readouts assessed by flow cytometry and immunohistochemistry; and objective response, duration of response, and progression-free survival (PFS) according to the RECIST version 1.1, as assessed by the investigator. Exploratory endpoints included the correlation of baseline and on-treatment biomarkers with response to lartesertib treatment as well as relative changes in total blood cell count, immune cells, and circulatory factors during exposure to lartesertib.

### PK analysis

The PK of lartesertib was characterized during dose escalation using an intensive PK sample collection schedule. Blood samples were collected at multiple timepoints during Cycle 1, including on Day 1 (pre-dose at 0 hours and post-dose at 0.5, 1, 2, 3, 4, 6, 8, and 12 hours) and Day 8 (pre-dose at 0 hours and post-dose at 1, 2, 3, 4, 6, 8, and 12 hours). PK parameters were calculated from individual plasma concentration–time data using non-compartmental methods.

### PD analysis

The PD analyses in blood were conducted using samples collected during Cycle 1 at the following time points: Day 1, pre-dose, and post-dose at 2, 4, and 6 hours; Day 2 at pre-dose and post-dose at 2 to 4 hours; and Day 22 at pre-dose and post-dose at 2 and 4 hours. These samples were analyzed to assess levels of the PD biomarker, γ-H2AX, a phosphorylated histone variant known to represent an early and sensitive marker of DSB response (18). Each sample was stimulated *ex vivo* with bleomycin, a DNA DSB inducer that activates ATM function, or with phosphate-buffered saline as a negative control. γ-H2AX was subsequently measured in the CD45^+^ lymphocyte fraction by flow cytometry.

The PD analysis in tumor tissue was not conducted because only one patient consented to undergo a paired biopsy procedure for the analysis of phosphorylated checkpoint kinase 2 expression. However, the samples were deemed not evaluable as they were collected from a bone lesion, which affected the execution of the immunohistochemistry assay.

### Biomarker analyses

Serial blood samples collected at each visit were tested for total blood cell counts and immune cell subsets (e.g., T cells, B cells, and NK cells) to evaluate immunophenotyping by flow cytometry.

Circulating immune factors (e.g., IFN-β, IFN-γ, IL-1β, IL-2, IL-4, IL-6, IL-8, IL-10, IL-12p70, IL-13, TNF-α, and NKG2DL) were assessed using immunofluorescence assays. Additionally, blood samples were tested for plasma ctDNA by next-generation sequencing at Guardant Health using the OMNI panel. This analysis was conducted to evaluate surrogate markers of tumor dynamics, sensitivity, and resistance to treatment. Molecular response (MR) was assessed and defined as strongest change of—per visit averaged—somatic variant allele frequency (VAF) against the pretreatment level as follows:

$$\text{Best}\;\text{delta}\;\text{mean}\;\text{VAF}\;=\;\mathrm{MIN}\;\left({\text{meanVAF}}_{\text{visiti}}\;–\;{\text{meanVAF}}_{\text{pretreatment}}\;/\;{\text{meanVAF}}_{\text{pretreatment}}\right)$$

### Statistical analyses

The DLT analysis set included all patients who received ≥80% of the planned cumulative dose during the DLT period (Period 1) and had completed the DLT period, or patients who experienced ≥1 DLT during the DLT period, regardless of the number of lartesertib doses administered. Additional details on all analysis sets are provided in the Supplementary Material S1. Analyses to determine dose escalation were performed on the DLT analysis set and were based on available safety and PK data. A Bayesian two-parameter logistic regression model (BLRM) was applied to assist the safety monitoring committee in dose recommendations during lartesertib dose escalation. For the determination of the MTD, posterior probabilities (2.5%, 25%, 50%, 75%, and 97.5% quantiles) for DLT rates at selected doses were estimated by a BLRM. The target toxicity of the model for MTD was set at 30%.

AEs and diseases were coded according to the Medical Dictionary for Regulatory Activities (RRID: SCR_003751) version 25.1 (until 30 days after the last study treatment). The severity of AEs was graded using the NCI Common Terminology Criteria for Adverse Events (RRID: SCR_010296) version 5.0.

For the efficacy analysis, the proportion of patients with objective response, defined as radiologic complete response (CR) + partial response, was tabulated, and the 95% confidence interval (CI) was estimated using the Clopper–Pearson method. PFS was estimated using the Kaplan–Meier method. The median PFS time and rate estimates at 3, 6, and 12 months, as well as every 6 months thereafter, were also summarized.

## Results

**Figure 1.**
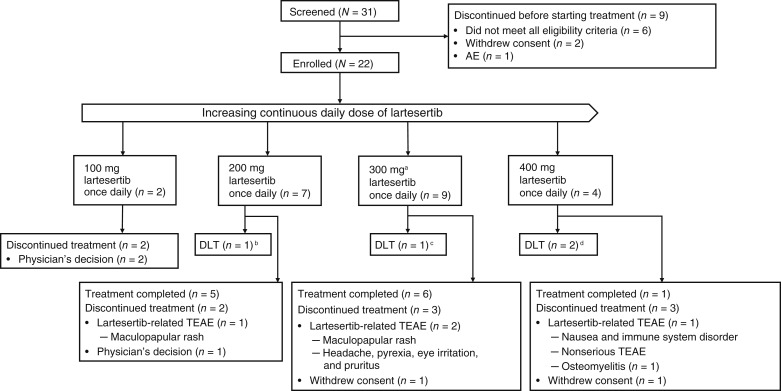
Patient disposition. ^a^declared as MTD; ^b^maculopapular rash grade 3 (*n* = 1); ^c^maculopapular rash grade 1 and pyrexia grade 2 (*n* = 1); and ^d^maculopapular rash grade 3 (*n* = 2). DLT, dose-limiting toxicity; MTD, maximum tolerated dose; RDE, recommended dose for expansion; TEAE, treatment-emergent adverse event.

From May 24, 2021, to December 12, 2022, 22 patients with advanced solid tumors were enrolled and treated in the study (Fig. 1). Baseline characteristics are summarized in Table 1. The median age was 58.5 years (range, 43–79), and 59.1% of patients were female. Overall, 54.5% of patients received three or more lines of prior anticancer therapy. The most common primary tumor types were endometrium/ovary (31.8%), prostate (18.2%), colon/rectum (13.6%), and pancreas (9.1%). The median duration of treatment was 5.64 weeks (range, 0.1–18.0).

**Table 1.** Patient demographics and baseline characteristics.

Demographic/baseline characteristics	Lartesertib monotherapy				
	100 mg *n* = 2	200 mg *n* = 7	300 mg *n* = 9	400 mg *n* = 4	Total *N* = 22
Age, years [median (min, max)]	74.5 (70, 79)	54.0 (43, 62)	61.0 (44, 70)	55.5 (53, 65)	58.5 (43, 79)
Female, *n* (%)	1 (50.0)	5 (71.4)	4 (44.4)	3 (75.0)	13 (59.1)
Race, *n* (%)	​	​	​	​	​
White	2 (100.0)	5 (71.4)	7 (77.8)	3 (75.0)	17 (77.3)
Other	0 (0.0)	2 (28.6)	2 (22.2)	1 (25.0)	5 (22.7)
ECOG PS	​	​	​	​	​
0	0 (0.0)	1 (14.3)	2 (22.2)	1 (25.0)	4 (18.2)
1	2 (100.0)	6 (85.7)	7 (77.8)	3 (75.0)	18 (81.8)
Time since initial cancer diagnosis, years [median, (min, max)]	2.75 (2.5, 3.0)	2.00 (0.7, 3.1)	4.20 (1.7, 14.4)	2.65 (1.4, 3.3)	2.85 (0.7, 14.4)
Time since documented locally advanced, inoperable, or metastatic disease diagnosis, years [mean (SD)]	2.75 (0.354)	1.35 (1.078)	5.73 (4.460)	2.20 (1.131)	3.42 (3.477)
Prior anticancer therapy regimens for metastasis of locally advanced disease, *n* (%)	​	​	​	​	​
1	0 (0.0)	2 (28.6)	0 (0.0)	1 (25.0)	3 (13.6)
2	1 (50.0)	1 (14.3)	0 (0.0)	0 (0.0)	2 (9.1)
3	1 (50.0)	1 (14.3)	3 (33.3)	0 (0.0)	5 (22.7)
≥4	0 (0.0)	3 (42.9)	2 (22.2)	2 (50.0)	7 (31.8)
Disease stage at study entry, *n* (%)	​	​	​	​	​
Stage 3	0 (0.0)	0 (0.0)	1 (11.1)	0 (0.0)	1 (4.5)
Stage 4	2 (100.0)	7 (100.0)	8 (88.9)	4 (100.0)	21 (95.5)
Site of primary tumor, *n* (%)	​	​	​	​	​
Colon/rectum	0 (0.0)	2 (28.6)	0 (0.0)	1 (25.0)	3 (13.6)
Endometrium/ovary	1 (50.0)	2 (28.6)	2 (22.2)	2 (50.0)	7 (31.8)
Lung	0 (0.0)	0 (0.0)	0 (0.0)	1 (25.0)	1 (4.5)
Nasopharynx	0 (0.0)	0 (0.0)	1 (11.1)	0 (0.0)	1 (4.5)
Pancreas	0 (0.0)	1 (14.3)	1 (11.1)	0 (0.0)	2 (9.1)
Parotid gland	0 (0.0)	0 (0.0)	1 (11.1)	0 (0.0)	1 (4.5)
Prostate gland	1 (50.0)	0 (0.0)	3 (33.3)	0 (0.0)	4 (18.2)
Retroperitoneum	0 (0.0)	1 (14.3)	0 (0.0)	0 (0.0)	1 (4.5)
Thyroid gland	0 (0.0)	0 (0.0)	1 (11.1)	0 (0.0)	1 (4.5)
Unknown primary site	0 (0.0)	1 (14.3)	0 (0.0)	0 (0.0)	1 (4.5)

Abbreviation: ECOG PS, Eastern Cooperative Oncology Group performance status; SD, standard deviation.

### DLTs and MTD

Overall, 18 of 22 patients (82%) were evaluable for DLTs. Of those, four patients (22.2%) in the DLT analysis set experienced a total of five DLTs (Fig. 1). Maculopapular rash was the most common DLT (four events in four patients; one each at DLs 200 mg and 300 mg and two at DL 400 mg). Pyrexia was reported in one patient at DL 300 mg. The BLRM suggested that the MTD of lartesertib monotherapy was 300 mg once daily, as this was the highest tested dose below the level with median DLT probability of 30%. This finding meets the MTD criteria, when administered continuously, as the median estimated DLT probability of 30% was within the range of 17% to 30%, and the upper boundary of the one-sided 95% credible interval was below the 40% threshold. No recommended dose for expansion for monotherapy was declared as further development of lartesertib is planned for combination therapy only.

### Safety outcomes

All 22 (100%) patients experienced at least one TEAE, whereas 15 (68.2%) patients experienced at least one lartesertib-related TEAE (Supplementary Table S2). The most frequently reported TEAEs were fatigue (*n* = 9; 40.9%), pyrexia (*n* = 8; 36.4%), and nausea and increased blood creatinine (*n* = 6; 27.3% each; Supplementary Fig. S1). The most common grade ≥3 TEAE was anemia observed in four patients (18.2%), followed by increased aspartate aminotransferase, decreased lymphocyte count, and maculopapular rash in three patients (13.6%) each. Four patients (18.2%) experienced lartesertib-related grade 3 TEAEs; decreased lymphocyte count and maculopapular rash were reported in one patient at DL 200 mg, anemia in one patient at DL 300 mg, maculopapular rash in one patient at DL 400 mg, and nausea and maculopapular rash in another patient at DL 400 mg. The last patient also experienced a TEAE of grade 4 immune system disorder (hypersensitivity). Lartesertib was permanently discontinued for the events of nausea and hypersensitivity. Additional details on the safety data are summarized in Supplementary Tables S3 and S4.

Serious TEAEs were reported in nine (40.9%) patients. Nausea (*n* = 2; 9.1%) and maculopapular rash (*n* = 2; 9.1%) were the most common serious TEAEs. Overall, five serious lartesertib-related TEAEs were reported in two patients (9.1%) at DL 400 mg, including two events of maculopapular rash (grade 3), one event of immune system disorder (hypersensitivity; grade 4), and one event each of nausea and pyrexia (grade 3). Most hematological TEAEs were assessed as non-serious, except for one event of grade 3 decreased lymphocyte count. Grade ≥3 anemia was reported in four patients (18.2%), and grade 3 decreased lymphocyte count was observed in three patients (13.6%). Hematological TEAEs of grade 1 or 2 in severity included decreased neutrophil count, platelet count, and white blood cell count, each occurring in one patient (4.5%).

TEAEs leading to permanent treatment discontinuation were reported in five patients, including grade 3 maculopapular rash (one patient at DL 200 mg); grade 3 urinary tract infection (one patient at DL 200 mg); grade 1 headache, pyrexia, eye irritation, and pruritus (one patient at DL 300 mg); grade 1 maculopapular rash (one patient at DL 300 mg); and grade 3 nausea and grade 4 immune system disorder (both events in one patient at DL 400 mg). Temporary treatment discontinuation due to TEAEs was reported in five patients, including grade 3 pneumonia and grade 2 gait disturbance (one patient at DL 200 mg); grade 1 pyrexia, grade 2 increased blood bilirubin, and hypotension (all in one patient at DL 200 mg); grade 3 increased blood bilirubin (one patient at DL 200 mg); grade 2 maculopapular rash (two episodes in one patient at DL 400 mg); and grade 1 pyrexia and grade 3 (maximum toxicity grade) maculopapular rash (one patient at DL 400 mg). No TEAEs led to death during the study period.

No clinically meaningful changes were observed in vital signs and triplicate electrocardiogram values from baseline to any post-dose values for any patient. Most biochemistry parameters remained within their normal range.

### MRs

**Figure 2.**
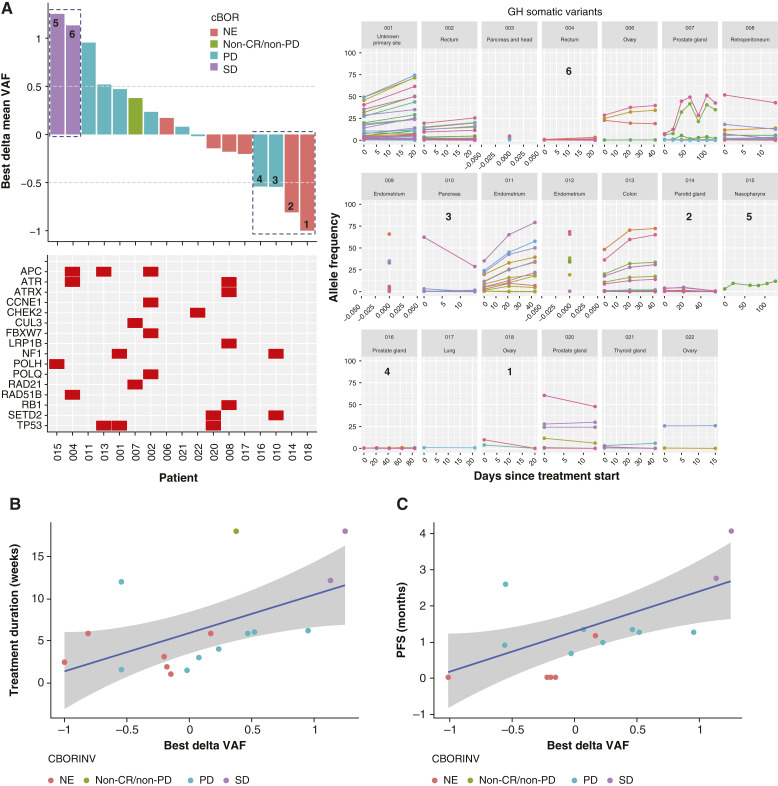
Correlation of MR with clinical response (**A**), treatment duration (**B**), and PFS (**C**). cBOR, confirmed best overall response; CBORINV, confirmed best overall response as assessed by investigator; CR, complete response; MR, molecular response; NE, not evaluable; PD, progressive disease; PFS, progression-free survival; PR, partial response; SD, stable disease; VAF, variant allele frequency.

MRs with a ctDNA VAF decrease of ≥50% from baseline were observed in four patients, but only a few genetic alterations were found in these patients. MR, after an initial VAF increase, correlated with disease stabilization in two patients (Fig. 2A). In ctDNA analysis, a mutation in the *POLH* gene was detected in one patient with stable disease (SD), whereas mutations in *APC*, *ATR*, and *RAD51B* genes were detected in another patient with SD. DDR-relevant genetic alterations were detected in *TP53* (*n* = 3), *APC* (*n* = 3), *ATR* (*n* = 2), *ATRX* (*n* = 1), and 14 other genes. Few somatic variants were reported for eight of 21 patients. The full panel of mutations from the Guardant Health OMNI panel is provided in Supplementary Table S5. Additionally, MRs did not correlate with treatment duration and PFS (Fig. 2B and C).

### PK and PD analyses

All 22 patients (100%) were included in the PK analysis. Lartesertib was rapidly absorbed, with a median time to reach maximum observed plasma concentration (t_max_) ranging from approximately 1 to 2 hours post-dose and a geometric mean elimination half-life (t_1/2_) ranging from approximately 5 to 7 hours across the reported groups on both Day 1 and Day 8 (Supplementary Fig. S2). Exposure increased in a dose-dependent manner, with minor accumulation after multiple once daily doses as indicated by geometric mean accumulation ratios R_acc(AUCτ)_ ranging from 1.15 to 1.29 and R_acc(Cmax)_ ranging from 1.10 to 1.18.

Eight patients (36%) were evaluable for PD analysis. A trend of increasing lartesertib target inhibition of the biomarker γ-H2AX was observed at 2, 4, and 6 hours after first treatment on Day 1 and was sustained on Day 2 at 2 to 4 hours across all four DLs, without a clear evidence of dose correlation (Supplementary Fig. S3).

### Efficacy

**Figure 3.**
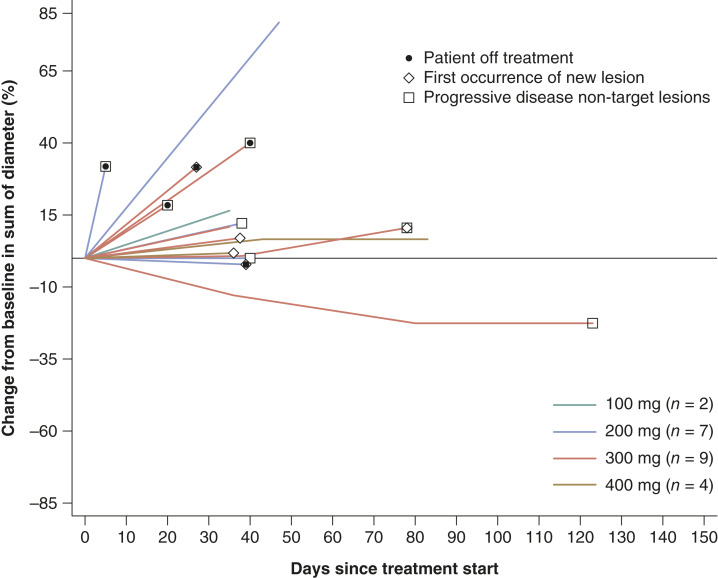
Tumor burden changes. Of the 22 patients treated, 15 (68.2%) patients were evaluable for response.

Of the 22 patients, 15 patients (68.2%) were evaluable for tumor response. Two patients (9.1%) with nasopharyngeal and pancreatic head cancer, respectively, had SD as the best overall response. Notably, the patient with nasopharyngeal cancer experienced SD for 120 days. Twelve patients (54.5%) had progressive disease, and one patient (4.5%) with prostate gland cancer had non-CR/non–progressive disease (Fig. 3; Supplementary Table S6). The median PFS was 1.3 (95% CI, 0.99–1.58) months. All PFS events, except in one patient, were attributed to disease progression. Among the five deaths reported during the follow-up period, progressive disease led to deaths in three patients, whereas the cause of death of the other two patients was unknown.

### Immunophenotyping and circulatory factors

Lartesertib treatment did not cause any significant change in the levels of most explored immune cell subsets at the tested DLs, including myeloid-derived suppressor cells and T and B lymphocytes, with proliferating (Ki67+) subsets included. A negative trend was observed in NK cell subsets across all DLs (Supplementary Fig. S4). No relevant trends in cytokine modulation (IFN-γ, IL-10, IL-6, IL-8, and TNF-α) were observed among different lartesertib DLs; only anecdotal variations were noted (Supplementary Fig. S5). Abnormally high baseline values of IFN-γ, IL-6, and TNF-α were observed in one patient at DL 400 mg who experienced treatment-emergent grade 3 nausea and grade 4 immune system disorder (hypersensitivity).

## Discussion

Part 1A of the DDRiver Solid Tumors 410 study determined the MTD of lartesertib monotherapy at 300 mg once daily. Overall, five DLTs were reported in four patients, with maculopapular rash being the most common DLT. Fatigue was the most frequently reported TEAE, whereas the most common grade ≥3 TEAE was anemia. No treatment-related deaths were observed during this study. Across the analyzed DLs (100–400 mg once daily), lartesertib was rapidly absorbed following oral administration with median t_max_ between ∼1 to 2 hours post-dose and geometric mean t_1/2_ ranging from ∼5 to 7 hours on both Day 1 and Day 8. Exposure increased in a dose-related manner across the dose groups after multiple once daily doses with minor accumulation. The target exposure and engagement were achieved without significant hematological toxicities. PD analyses showed a trend of reduction in γ-H2AX levels from Day 1 to Day 2, reaching 80% to 100% target inhibition on Day 2 with the dose range 100–400 mg once daily without clear evidence of dose dependence. This analysis is limited because of a small PD dataset. As PD was a secondary endpoint of this study, data quality criteria were established during PD biomarker assay development for data acceptability. This led to the exclusion of 10 patients whose baseline samples did not pass these criteria (five patients because of insufficient event counts and five patients because of bleomycin γ-H2AX induction failure). In addition, three patients were excluded because the baseline sample was not collected, and one patient was excluded because the sample was collected after treatment administration.

No significant persistent effects of lartesertib on the immune system were detected. Considering the limited sample size and high variability within each subpopulation, lartesertib treatment did not cause any consistent changes in the levels of most immune cell subsets across all tested DLs. MRs were observed in four patients; however, no correlation was observed with median treatment duration or PFS. This lack of correlation may be due to the low allele frequencies observed in several ctDNA samples, as well as the small number of trackable somatic variants present in the limited number of cases, which limit the robustness of these measurements. No relevant trend was observed in cytokine modulation among different lartesertib DLs.

As expected, limited clinical activity was observed for lartesertib monotherapy in this dose-escalation study conducted in an unselected, heavily pre-treated patient population with advanced-stage disease refractory to standard treatments.

ATM inhibition impairs the cellular DSB repair via homologous recombination, leading tumor cells to depend on remaining functional compensatory DDR pathways, such as ATR (16, 19). Simultaneous inhibition of ATM and ATR has been shown to induce cancer cell death through synthetic lethality in preclinical models (19). In a recent study using patient-derived xenograft models of triple-negative breast cancer, lartesertib in combination with the ATR inhibitor gartisertib led to a substantially improved tumor control rate compared with single-agent gartisertib (42% vs. 27%; 17). Based on this, we hypothesize that the dual inhibition of ATM and ATR could enhance anticancer efficacy and improve clinical outcomes, supporting the further assessment of lartesertib in combination with an ATR inhibitor (20).

Initial data reported from the phase Ib open-label trial DDRiver Solid Tumors 320 (NCT05396833) in patients with advanced solid tumors support the rationale for the combination of lartesertib and the oral ATR inhibitor tuvusertib, with six of 42 analyzed patients experiencing SD for >16 weeks (21, 22). The regimen of tuvusertib 180 mg once daily + lartesertib 150 mg once daily, 2 weeks on/2 weeks off, was selected for investigation in expansion cohorts in patients with prostate and endometrial cancers (21, 23). Additionally, DDRiver EOC 302 (NCT06433219), a randomized phase II study, is currently evaluating lartesertib in combination with tuvusertib in patients with advanced epithelial ovarian cancer (24, 25).

In conclusion, lartesertib was well tolerated in patients with advanced solid tumors. Target exposure and engagement were achieved without significant hematological toxicities. Furthermore, no significant effects of lartesertib on the immunophenotype, MR, and circulatory factors were observed. Our findings support the potential for lartesertib as a combination partner with another DDR inhibitor, a hypothesis that is currently being evaluated in ongoing clinical trials.

## Supplementary Material

Supplementary Material 1 Supplementary Material

Supplementary Table S1 Representativeness of study participants

Supplementary Table S2 Safety overview

Supplementary Table S3 Most common TEAE (reported in ≥10% of patients, overall) by primary system organ class and preferred term – Safety analysis set

Supplementary Table S4 TEAEs by worst NCI-CTCAE severity Grade, primary system organ class and preferred term – Full analysis set/Safety analysis set

Supplementary Table S5 Full panel of mutations from Guardant OMNI panel

Supplementary Table S6 Efficacy overview

Supplementary Figure S1 Supplementary Figure S1

Supplementary Figure S2 Supplementary Figure S2

Supplementary Figure S3 Supplementary Figure S3

Supplementary Figure S4 Supplementary Figure S4

Supplementary Figure S5 Supplementary Figure S5

## Data Availability

Any requests for data by qualified scientific and medical researchers for legitimate research purposes will be subject to the healthcare business of Merck KGaA Darmstadt, Germany’s (CrossRef funder ID: 10.13039/100009945) Data-Sharing Policy. All requests should be submitted in writing to the healthcare business of Merck KGaA Darmstadt, Germany’s data-sharing portal (https://www.emdgroup.com/en/research/our-approach-to-research-and-development/healthcare/clinical-trials/commitment-responsible-data-sharing.html). When the healthcare business of Merck KGaA Darmstadt, Germany, has a co-research, co-development, or co-marketing or co-promotion agreement, or when the product has been out-licensed, the responsibility for disclosure might be dependent on the agreement between parties. Under these circumstances, the healthcare business of Merck KGaA Darmstadt, Germany, will endeavor to gain agreement to share data in response to requests.
