# Supplementary material for: A First-in-Human Study of ATM Inhibitor Lartesertib as Monotherapy in Patients with Advanced Solid Tumors
Source: Clin Cancer Res. 2025 Aug 28;31(21):4429–37. doi: 10.1158/1078-0432.CCR-25-1648 (PMC12580772; doi:10.1158/1078-0432.CCR-25-1648)
Supplement: Supplementary Material 1 — Supplementary Material [file ccr-25-1648_supplementary_material_1_suppms1.docx]

**A First-in-Human Study of ATM Inhibitor Lartesertib as Monotherapy in Patients with Advanced Solid Tumors**

**Supplementary Material**

# **Study Design**

Part 1A was a lartesertib monotherapy dose-escalation designed to determine the safety, tolerability, pharmacokinetics (PK), pharmacodynamics (PD), and early signs of efficacy in participants with solid tumors. For each patient, the study included a Screening period lasting up to 28 days and the estimated total duration in the study was an approximate of 105 days/15 weeks. Patients were treated with lartesertib orally as a tablet, at doses ranging from 100 to 400 mg once daily (QD) under a fasting condition of 2 hours pre-dose to 1-hour post-dose in general, and 2 hours pre-dose to 4 hours post-dose on days where PK-time matched triplicate ECG were collected. Patients were treated until disease progression, death, adverse events (AEs) leading to discontinuation of study intervention(s), or withdrawal of consent, whichever occurred first, or end of study. There was an end of study intervention visit (within 7 days after last dose), and a safety follow-up period of 30 days (±7 days) after the last study intervention intake. A patient who experienced an event of progressive disease or death was considered as having completed the study. A patient who discontinued from study without progressive disease but due to an AE, withdrawal, or any other reason, was considered as having discontinued from the study.

# **Methods**

## **Inclusion Criteria:**

Participants are eligible to be included in the study only if all the following criteria apply:

Age

1. Are ≥ 18 years of age at the time of signing the informed consent.

Type of Participant and Disease Characteristics (including organ function)

1. Patients with advanced solid tumors, for whom no standard of care therapy exists or for whom is not considered sufficiently effective, or who cannot tolerate standard of care.
2. Eastern Cooperative Oncology Group Performance status 0 or 1.
3. Adequate hematological function defined by absolute neutrophil count, ≥ 1.5 × 109/L, platelet count ≥ 100 × 109/L, and hemoglobin ≥ 9 g/dL, and without growth factor treatment or blood transfusion within 2 weeks before the study intervention start.
4. Adequate hepatic function defined by total bilirubin level ≤ 1.5 × upper limit of normal (ULN), aspartate aminotransferase level ≤ 3 × ULN, and an alanine aminotransferase (ALT) level ≤ 3 × ULN. For patients with liver metastases, aspartate aminotransferase ≤ 5.0 × ULN, ALT ≤ 5.0 × ULN, and total bilirubin ≤ 3.0× ULN is acceptable. For patients with Gilbert’s disease, total bilirubin ≤ 2.0 mg/dL or direct bilirubin ≤ 1 × ULN is acceptable.
5. Adequate renal function defined by an estimated glomerular filtration rate > 60 mL/min according to the Cockcroft-Gault equation: (Glomerular filtration rate = {((140–age) x weight)/(72xSCr)} x 0.85 (if female).

Sex

1. Are male and/or female
2. Contraceptive use by males or females will be consistent with local regulations on contraception methods for those participating in clinical studies.

Male participants:

Agree to the following during the study intervention period and for at least 3 months after the last dose of study intervention:

- Refrain from donating sperm.

PLUS, either:

- Abstain from any activity that allows for exposure to ejaculate.

OR

- Use a male condom:

When having sexual intercourse with a woman of childbearing potential (WOCBP) and advise her to use a highly-effective contraceptive method with a failure rate of
< 1% per year, since a condom may break or leak.

When engaging in any activity that allows for exposure to ejaculate to another person.

- Male participants must use a male condom with pregnant female partners during the study.

Female participants:

Are not pregnant or breastfeeding, and at least 1 of the following conditions applies:

- Not a WOCBP

OR

- If a WOCBP, use a highly effective contraceptive method (i.e., with a failure rate of < 1% per year), preferably with low user dependency, for the following time periods:
  - Before the first dose of the study intervention(s), if using hormonal contraception:
    - Has completed at least one 4-week cycle of an oral contraception pill and either had or has begun her menses

OR

- Has used a depot contraceptive or extended cycle oral contraceptive for least 28 days and has a documented negative pregnancy test using a highly sensitive assay.
- During the intervention period
- After the study intervention period (i.e., after the last dose of study intervention is administered) for at least 6 months after the last dose of study intervention and agree not to donate eggs (ova, oocytes) for reproduction during this period

The Investigator evaluates the effectiveness of the contraceptive method in relationship to the first dose of study intervention

- Have a negative urine pregnancy test, as required by local regulations, within 24 hours before the first dose of study intervention
- Women should not breastfeed during the study and for at least 1 month after the study period, (i.e., after the last dose of study intervention is administered).

The Investigator reviews the medical history, menstrual history, and recent sexual activity to decrease the risk for inclusion of a female with an early undetected pregnancy.

Informed Consent

1. Capable of giving signed informed consent, which includes compliance with the requirements and restrictions listed in the informed consent form (ICF) and the study protocol.

**Exclusion Criteria:**

Participants are excluded from the study if any of the following criteria apply:

Medical Conditions

1. Clinically significant (i.e., active) uncontrolled intercurrent illness including, but not limited to:

- Active infection (i.e., requiring systemic antibiotics or antifungals)
- Uncontrolled arterial hypertension (i.e., systolic blood pressure > 150 mmHg, diastolic blood pressure > 100 mmHg)
- Symptomatic congestive heart failure (≥ New York Heart Association Classification Class II), unstable angina, myocardial infarction or a coronary revascularization procedure within 180 days of study entry
- Calculated average QT interval corrected using Fridericia’s formula (QTcF) of > 450 msec for males and > 470 msec for females.
- A history of additional risk factors for Torsades de Pointes (e.g., clinically relevant hypokalemia, family history of Long QT Syndrome)
- The use of concomitant medications that prolong the QT/QTc interval
- Severe cardiac arrhythmia requiring medication
- Cerebral vascular accident/stroke
- Clinically significant liver disease consistent with Child Pugh Class B or C; and/or
- Any psychiatric illness/social situations that would limit compliance with study requirements.

1. Presence of brain metastases unless clinically stable (without evidence of progression by imaging for at least four weeks prior to the first dose of study intervention and any neurologic symptoms have returned to baseline), no evidence of new brain metastases, and on a stable or decreasing dose or without steroids for at least 14 days prior to study intervention. Participants with carcinomatous meningitis are excluded regardless of clinical stability. Central nervous system imaging at Screening is not mandatory.
2. Has a known additional malignancy that is progressing and/or requires active treatment. In addition, participants are excluded who were diagnosed with another malignancy within 3 years of starting study intervention. Exceptions include fully resected basal cell carcinoma of the skin or squamous cell carcinoma of the skin, in situ cervical cancer, fully resected ductal carcinoma in situ, and Stage IA noninvasive grade I endometrioid endometrial cancer, that has undergone curative therapy. Participants with other localized malignancies treated with curative intent need to be discussed with the Medical Monitor. Participant must not have any known history of myelodysplastic syndrome or acute myeloid leukemia.
3. Has known ataxia telangiectasia
4. Participants with tumors harboring previously identified ATM mutations
5. Individuals with known human immunodeficiency virus and/or active viral hepatitis (B and/or C), and individuals on viral hepatitis B therapy are excluded. However, individual with Hepatitis C treated with curative therapy are not considered actively infected.
6. Has a history or current evidence of any condition, therapy, and/or laboratory abnormality that might confound the results of the trial, interfere with the participant’s participation for the full duration of the trial, and/or is not in the best interest of the participant to participate, in the opinion of the treating investigator.

Gastrointestinal Conditions

1. Serious gastrointestinal bleeding within 3 months, refractory nausea and vomiting, uncontrolled diarrhea, known malabsorption, significant small bowel resection or gastric bypass surgery, use of feeding tubes, other chronic gastrointestinal disease (including exocrine pancreatic insufficiency requiring pancreatic enzyme replacement therapy) and/or other situation that may preclude adequate absorption of oral medications

Prior/Concomitant Therapy

1. Participants who may have received any of the following anticancer therapy(ies), or concomitant medications within the following windows per the first day of study intervention administration:
   - Hematopoietic growth factors (including erythropoietin, darbepoetin, granulocyte colony stimulating factor, granulocyte macrophage-colony stimulating factor, and platelet stimulators [e.g. eltrombopag, romiplostim, or IL-11]) and blood transfusions within 2 weeks.
   - Anticancer treatment within 28 days or 5 half-lives, whichever is shorter, except for nitrosoureas or mitomycin C (within 6 weeks).
   - Participants receiving prior treatment with an ATM inhibitor.
   - Prior curative-intent radiotherapy within 4 weeks. Prior palliative radiotherapy to metastatic lesion(s) is permitted provided it was completed > 2 weeks prior to study enrollment and toxicities have recovered to Grade ≤ 1.
   - Major surgery within 4 weeks and not recovered for side effects Grade ≤ 1, according to investigator’s judgment.
   - Any other type of anticancer therapy, not listed above, within 4 weeks.
   - Strong inhibitors or inducers of CYP3A4 or P-gp that cannot be discontinued as outlined in Section 6.5.3. Examples of strong inhibitors of CYP3A4 and P-gp inhibitors or strong inducers of CYP3A are provided in Table 9.
   - Additional excluded concomitant medications include irinotecan, oral direct thrombin inhibitors, and digoxin.
   - Concomitant treatment with proton pump inhibitors is prohibited. The administration of H2-receptor antagonists will be permitted as long as they are not administered for 12 hours before or 2 hours after the dosing of lartesertib. Antacids cannot be administered 2 hours before or 2 hours after the dosing of lartesertib.
2. Participants not recovered from AEs (i.e., Grade ≤ 1) of prior anticancer therapies. Exception: Grade 2 AEs not constituting a safety risk, based on the Investigator’s judgement, must be consulted with the Sponsor prior to enrolment.

Prior/Concurrent Clinical Study Experience

1. Is currently participating and receiving study therapy or has participated in a study of an investigational agent and received study therapy and/or used an investigational device within 4 weeks of the first lartesertib administration of study intervention or longer if described elsewhere.
2. Hypersensitivity to the active substance or to any of the excipients of lartesertib.

Other Exclusions

1. Pregnant or breastfeeding.

**Objectives**

The primary objective was to determine dose toxicity relationship and maximum tolerated dose (if reached) of lartesertib monotherapy in patients with advanced solid tumors. Secondary objectives were to determine the recommended dose for expansion, characterize the PK profile, preliminary clinical activity parameters of lartesertib monotherapy and assess the changes in PD markers of lartesertib activity in tumor and blood. Exploratory objectives were to evaluate molecular and morphological biomarkers of response in tumor tissue and in plasma, and to explore the potential impact on the immune system during lartesertib monotherapy.

**Analysis sets**

Overall, 4 analysis sets were defined in the study.

1. Full analysis set/safety analysis set (FAS/SAF) included all patients who received ≥1 dose of lartesertib.
2. Dose-limiting toxicity (DLT) analysis set included all patients who received ≥80% of the planned cumulative dose during the DLT period (Period 1) and have completed the DLT period or experienced ≥1 DLT during the DLT period, regardless of the number of lartesertib doses administered.
3. PK analysis set included all patients who were administered ≥1 dose of lartesertib and provided ≥1 measurable post-dose concentration.
4. PD analysis set included all patients who were administered ≥1 dose of lartesertib, had no clinically important protocol deviations/violations or events that would affect PD, and provided the baseline and ≥1 post-dose measurable PD endpoint.
