# Supplementary material for: A First-in-Human Study of ATM Inhibitor Lartesertib as Monotherapy in Patients with Advanced Solid Tumors
Source: Clin Cancer Res. 2025 Aug 28;31(21):4429–37. doi: 10.1158/1078-0432.CCR-25-1648 (PMC12580772; doi:10.1158/1078-0432.CCR-25-1648)
Supplement: Supplementary Table S1 — Representativeness of study participants [file ccr-25-1648_supplementary_table_s1_suppts1.docx]

**Supplementary Table S1: Representativeness of study participants**

| **Cancer type/subtype** | Advanced solid tumors |
| --- | --- |
| Considerations related to: | |
| Sex | Globally, an estimated 18.74 million cancer cases occurred in 2022, with  9.56 million (51%) cases occurring in men and 9.17 million (49%) cases occurring in women.^1^  In 2025, it is estimated that 1.05 million new cases of cancer will occur in male patients and 0.98 million new cases in female patients in the United States. Among these new cases, over 90% are expected to be solid tumors for both males and females.^2^ |
| Age | The median age at diagnosis of any cancer in the U.S. is 67 years. Cancer is most frequently diagnosed in individuals aged 65 to 74 years. The median age at death from cancer is 73 years.^3^ |
| Race/ethnicity | Between 2017 to 2021 in the United States, cancer incidence rates were highest among American Indian/Alaska Native populations, followed by White and Black populations, and lowest among Asian American/Pacific Islander populations.^2^ |
| Geography | Cancer ranks as the second most common cause of death worldwide.^4^ In the United States, it is the leading cause of death among men aged 60–79 years and women aged 40–79 years.^2^ |
| Overall representativeness of this study | The DDRiver Solid Tumors 410 study (NCT04882917) included 22 patients with advanced solid tumors. The proportion of female patients with cancer (59.1%) was higher compared to the reported global average of 49% female patients with cancer.^1^  The median patient age in our study was 58.5 years (range: 43 to 79), which is similar to the median age of 59 years of participants in phase 1 clinical trials in the U.S. recently reported for the period from 2000 to 2018.^5^  The most common primary tumor types were prostate (18.2%), ovary (18.2%), endometrium (13.6%), colorectal (13.6%) and pancreas (9.1%). In this respect, the patient population in our study differs from the global cancer population, where trachea, bronchus and lung cancer (13.2%), breast (12.2%), and colorectal cancer (10.3%) are most common.^1^  Ethnicities of patients included in this study were 77.3% White, 9.1% Asian, 4.5% Black or African American, and 9.1% other. For comparison, phase 1 clinical trial participants in the U.S. between 2000 and 2018 were 86.1% White, 4.5% Asian and 6.2% Black.^5^ This study was conducted at 2 sites in the US and at 1 site in Canada, which may limit racial and ethnic representations when compared with populations from other geographic areas. |

**References**

1. World Cancer Research Fund International: Worldwide cancer data. Available at [Worldwide cancer data | World Cancer Research Fund International (wcrf.org)](https://www.wcrf.org/preventing-cancer/cancer-statistics/worldwide-cancer-data/) [accessed 25 April 2025]
2. Siegel RL, Kratzer TB, Giaquinto AN, Sung H, Jemal A. Cancer statistics, 2025. CA Cancer J Clin. 2025 Jan-Feb;75(1):10-45. doi: 10.3322/caac.21871
3. National Cancer Institute Surveillance, Epidemiology and End Results (SEER) program. Cancer Stat Facts: Cancer of Any Site. Available at [Cancer of Any Site — Cancer Stat Facts](https://seer.cancer.gov/statfacts/html/all.html) [accessed 25 April 2023]
4. GBD 2015 Mortality and Causes of Death Collaborators. Lancet. 2016 Oct 8;388(10053):1459-1544. doi: 10.1016/S0140-6736(16)31012-1. Erratum in: Lancet. 2017 Jan 7;389(10064):e1. doi: 10.1016/S0140-6736(16)32605-8.
5. Dunlop H, et al. JAMA Netw Open. 2022;5(11):e2239884. doi:10.1001/jamanetworkopen.2022.39884
