# Supplementary material for: A First-in-Human Study of ATM Inhibitor Lartesertib as Monotherapy in Patients with Advanced Solid Tumors
Source: Clin Cancer Res. 2025 Aug 28;31(21):4429–37. doi: 10.1158/1078-0432.CCR-25-1648 (PMC12580772; doi:10.1158/1078-0432.CCR-25-1648)
Supplement: Supplementary Table S2 — Safety overview [file ccr-25-1648_supplementary_table_s2_suppts2.docx]

**Supplementary Table S2: Safety overview**

| **Number of Patients, n (%)** | **Lartesertib Monotherapy** | | | | |
| --- | --- | --- | --- | --- | --- |
|  | **100 mg n = 2** | **200 mg n = 7** | **300 mg n = 9** | **400 mg n = 4** | **Total N = 22** |
| **Any TEAE** | 2 (100.0) | 7 (100.0) | 9 (100.0) | 4 (100.0) | 22 (100.0) |
| Grade ≥3 | 0 (0.0) | 6 (85.7) | 4 (44.4) | 3 (75.0) | 13 (59.1) |
| Grade ≥4 | 0 (0.0) | 0 (0.0) | 0 (0.0) | 1 (25.0) | 1 (4.5) |
| **Any lartesertib-related TEAE** | 1 (50.0) | 2 (28.6) | 8 (88.9) | 4 (100.0) | 15 (68.2) |
| Grade ≥3 | 0 (0.0) | 1 (14.3) | 1 (11.1) | 2 (50.0) | 4 (18.2) |
| Grade ≥4 | 0 (0.0) | 0 (0.0) | 0 (0.0) | 1 (25.0) | 1 (4.5) |
| **Any serious TEAE** | 0 (0.0) | 4 (57.1) | 2 (22.2) | 3 (75.0) | 9 (40.9) |
| **Any lartesertib-related serious TEAE** | 0 (0.0) | 0 (0.0) | 0 (0.0) | 2 (50.0) | 2 (9.1) |
| **Any AESI cytopenia^a^** | 0 (0.0) | 1 (14.3) | 2 (22.2) | 1 (25.0) | 4 (18.2) |
| Grade ≥3 (all anemia events) | 0 (0.0) | 1 (14.3) | 2 (22.2) | 1 (25.0) | 4 (18.2) |
| **Any TEAE leading to temporary treatment discontinuation** | 0 (0.0) | 3 (42.9) | 0 (0.0) | 2 (50.0) | 5 (22.7) |
| **Any TEAE leading to permanent treatment discontinuation** | 0 (0.0) | 2 (28.6) | 2 (22.2) | 1 (25.0) | 5 (22.7) |
| **Any TEAE leading to death** | 0 (0.0) | 0 (0.0) | 0 (0.0) | 0 (0.0) | 0 (0.0) |
| **Any lartesertib-related TEAE leading to death** | 0 (0.0) | 0 (0.0) | 0 (0.0) | 0 (0.0) | 0 (0.0) |

^a^Includes lymphopenia, leukopenia/neutropenia, thrombocytopenia, anemia (Grade ≥3 or with complications)
AESI, adverse event of special interest; TEAE, treatment-emergent adverse event; TRAE, treatment-related adverse event
