# Supplementary material for: A First-in-Human Study of ATM Inhibitor Lartesertib as Monotherapy in Patients with Advanced Solid Tumors
Source: Clin Cancer Res. 2025 Aug 28;31(21):4429–37. doi: 10.1158/1078-0432.CCR-25-1648 (PMC12580772; doi:10.1158/1078-0432.CCR-25-1648)
Supplement: Supplementary Table S3 — Most common TEAE (reported in ≥10% of patients, overall) by primary system organ class and preferred term – Safety analysis set [file ccr-25-1648_supplementary_table_s3_suppts3.docx]

**Supplementary Table S3: Most common TEAE (reported in ≥10% of patients, overall) by primary system organ class and preferred term – Safety analysis set**

| **Number of Patients, n (%)** | **Lartesertib Monotherapy** | | | | |
| --- | --- | --- | --- | --- | --- |
|  | **100 mg n = 2** | **200 mg n = 7** | **300 mg n = 9** | **400 mg n = 4** | **Total N = 22** |
| **TEAEs reported in ≥10% of the ‘Total’ patient group** | | | | | |
| Anemia | 0 (0.0) | 1 (14.3) | 2 (22.2) | 1 (25.0) | 4 (18.2) |
| Abdominal pain | 0 (0.0) | 3 (42.9) | 1 (11.1) | 0 (0.0) | 4 (18.2) |
| Diarrhea | 1 (50.0) | 0 (0.0) | 1 (11.1) | 2 (50.0) | 4 (18.2) |
| Nausea | 1 (50.0) | 2 (28.6) | 2 (22.2) | 1 (25.0) | 6 (27.3) |
| Vomiting | 1 (50.0) | 2 (28.6) | 1 (11.1) | 1 (25.0) | 5 (22.7) |
| Fatigue | 0 (0.0) | 2 (28.6) | 4 (44.4) | 3 (75.0) | 9 (40.9) |
| Pyrexia | 0 (0.0) | 2 (28.6) | 4 (44.4) | 2 (50.0) | 8 (36.4) |
| Urinary tract infection | 0 (0.0) | 1 (14.3) | 1 (11.1) | 2 (50.0) | 4 (18.2) |
| Alanine aminotransferase increased | 0 (0.0) | 1 (14.3) | 2 (22.2) | 1 (25.0) | 4 (18.2) |
| Aspartate aminotransferase increased | 0 (0.0) | 2 (28.6) | 1 (11.1) | 1 (25.0) | 4 (18.2) |
| Blood bilirubin increased | 0 (0.0) | 3 (42.9) | 0 (0.0) | 0 (0.0) | 3 (13.6) |
| Blood creatinine increased | 0 (0.0) | 1 (14.3) | 2 (22.2) | 3 (75.0) | 6 (27.3) |
| Lymphocyte count decreased | 0 (0.0) | 1 (14.3) | 1 (11.1) | 1 (25.0) | 3 (13.6) |
| Maculopapular rash | 0 (0.0) | 1 (14.3) | 2 (22.2) | 2 (50.0) | 5 (22.7) |
| Headache | 0 (0.0) | 1 (14.3) | 1 (11.1) | 1 (25.0) | 3 (13.6) |
| Decreased appetite | 0 (0.0) | 2 (28.6) | 1 (11.1) | 0 (0.0) | 3 (13.6) |
| **Lartesertib-related TEAEs reported in ≥10% of the ‘Total’ patient group** | | | | | |
| Diarrhea | 1 (50.0) | 0 (0.0) | 1 (11.1) | 2 (50.0) | 4 (18.2) |
| Nausea | 1 (50.0) | 1 (14.3) | 2 (22.2) | 1 (25.0) | 5 (22.7) |
| Pyrexia | 0 (0.0) | 1 (14.3) | 3 (33.3) | 2 (50.0) | 6 (27.3) |
| Aspartate aminotransferase increased | 0 (0.0) | 1 (14.3) | 1 (11.1) | 1 (25.0) | 3 (13.6) |
| Blood creatinine increased | 0 (0.0) | 0 (0.0) | 1 (11.1) | 2 (50.0) | 3 (13.6) |
| Headache | 0 (0.0) | 1 (14.3) | 1 (11.1) | 1 (25.0) | 3 (13.6) |
| Maculopapular rash | 0 (0.0) | 1 (14.3) | 2 (22.2) | 2 (50.0) | 5 (22.7) |

TEAE, treatment-emergent adverse event
