# Supplementary material for: A First-in-Human Study of ATM Inhibitor Lartesertib as Monotherapy in Patients with Advanced Solid Tumors
Source: Clin Cancer Res. 2025 Aug 28;31(21):4429–37. doi: 10.1158/1078-0432.CCR-25-1648 (PMC12580772; doi:10.1158/1078-0432.CCR-25-1648)
Supplement: Supplementary Table S4 — TEAEs by worst NCI-CTCAE severity Grade, primary system organ class and preferred term – Full analysis set/Safety analysis set [file ccr-25-1648_supplementary_table_s4_suppts4.docx]

**Supplementary Table S4: TEAEs by worst NCI-CTCAE severity Grade, primary system organ class and preferred term – Full analysis set/Safety analysis set**

| **Primary system organ class Preferred term** | **Grade ≥3 n (%)** | **Grade ≥4 n (%)** | **Grade ≥5 n (%)** |
| --- | --- | --- | --- |
| **Subjects with at least one event** | 13 (59.1) | 1 (4.5) | 0 (0.0) |
| **Blood and lymphatic system disorders** | 4 (18.2) | 0 (0.0) | 0 (0.0) |
| Anemia | 4 (18.2) | 0 (0.0) | 0 (0.0) |
| **Gastrointestinal disorders** | 3 (13.6) | 0 (0.0) | 0 (0.0) |
| Abdominal pain | 1 (4.5) | 0 (0.0) | 0 (0.0) |
| Nausea | 2 (9.1) | 0 (0.0) | 0 (0.0) |
| Vomiting | 1 (4.5) | 0 (0.0) | 0 (0.0) |
| **Immune system disorders** | 1 (4.5) | 1 (4.5) | 0 (0.0) |
| **Investigations** | 6 (27.3) | 0 (0.0) | 0 (0.0) |
| Alanine aminotransferase increased | 2 (9.2) | 0 (0.0) | 0 (0.0) |
| Aspartate aminotransferase increased | 3 (13.6) | 0 (0.0) | 0 (0.0) |
| Blood bilirubin increased | 1 (4.5) | 0 (0.0) | 0 (0.0) |
| Blood alkaline phosphatase increased | 1 (4.5) | 0 (0.0) | 0 (0.0) |
| Gamma-glutamyltransferase increased | 1 (4.5) | 0 (0.0) | 0 (0.0) |
| Lymphocyte count decreased | 3 (13.6) | 0 (0.0) | 0 (0.0) |
| **Metabolism and nutrition disorders** | 1 (4.5) | 0 (0.0) | 0 (0.0) |
| Decreased appetite | 1 (4.5) | 0 (0.0) | 0 (0.0) |
| Dehydration | 1 (4.5) | 0 (0.0) | 0 (0.0) |
| **Skin and subcutaneous tissue disorders** | 3 (13.6) | 0 (0.0) | 0 (0.0) |
| Maculopapular rash | 3 (13.6) | 0 (0.0) | 0 (0.0) |

NCI-CTCAE, National Cancer Institute Common Terminology Criteria for Adverse Events
