# Supplementary material for: A First-in-Human Study of ATM Inhibitor Lartesertib as Monotherapy in Patients with Advanced Solid Tumors
Source: Clin Cancer Res. 2025 Aug 28;31(21):4429–37. doi: 10.1158/1078-0432.CCR-25-1648 (PMC12580772; doi:10.1158/1078-0432.CCR-25-1648)
Supplement: Supplementary Table S6 — Efficacy overview [file ccr-25-1648_supplementary_table_s6_suppts6.docx]

**Supplementary Table S6: Efficacy overview**

|  | **Lartesertib Monotherapy** | | | | | |
| --- | --- | --- | --- | --- | --- | --- |
|  | **100 mg n = 2** | **200 mg n = 7** | **300 mg n = 9** | **400 mg n = 4** | **Total N = 22** | |
| **Best overall response, n (%)** |  |  |  |  | |  |
| Stable disease | 0 (0.0) | 0 (0.0) | 1 (11.1) | 1 (25.0) | 2 (9.1) | |
| Non-complete response/non-progressive disease | 1 (50.0) | 0 (0.0) | 0 (0.0) | 0 (0.0) | 1 (4.5) | |
| Progressive disease | 0 (0.0) | 5 (71.4) | 6 (66.7) | 1 (25.0) | 12 (54.5) | |
| Not evaluable | 1 (50.0) | 2 (28.6) | 2 (22.2) | 2 (50.0) | 7 (31.8) | |
| **Progressive disease/Deaths, n (%)^a^** | 0 (0.0) | 6 (85.7) | 7 (77.8) | 1 (25.0) | 14 (63.6) | |
| **PFS, median (min, max), months** | NC (1.2,4.0) | 1.1 (0.0, 1.6) | 1.3 (0.0, 4.1) | NC (0.0, 2.8) | 1.3 (0.0, 4.1) | |

^a^These are PFS events and not the reason for study completion.

NC, noncalculable; PFS, progression-free survival
