# Supplementary figures and images for: A First-in-Human Study of ATM Inhibitor Lartesertib as Monotherapy in Patients with Advanced Solid Tumors
Source: Clin Cancer Res. 2025 Aug 28;31(21):4429–37. doi: 10.1158/1078-0432.CCR-25-1648 (PMC12580772; doi:10.1158/1078-0432.CCR-25-1648)

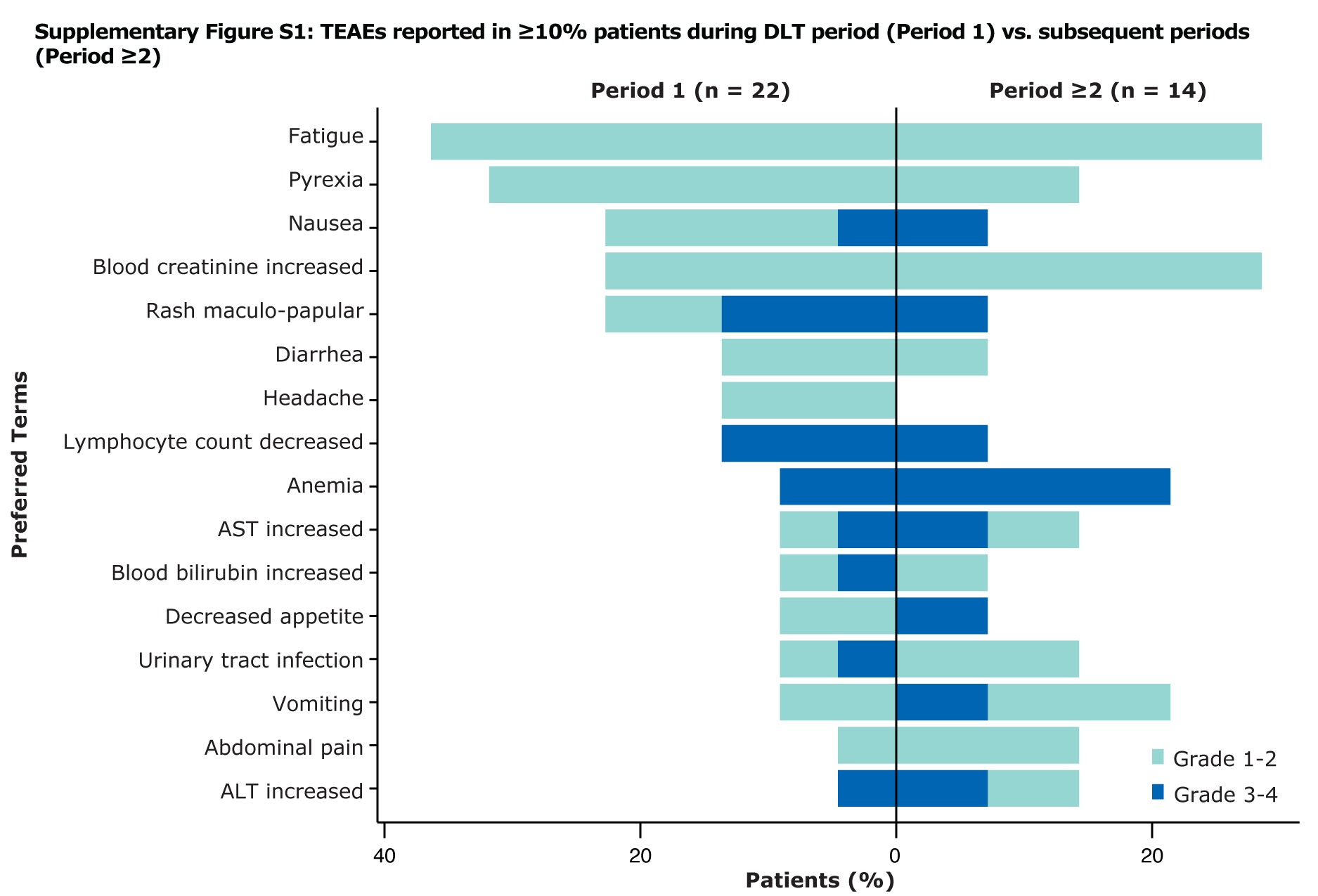

Supplement: Supplementary Figure S1 [file ccr-25-1648_supplementary_figure_s1_suppfs1.png]

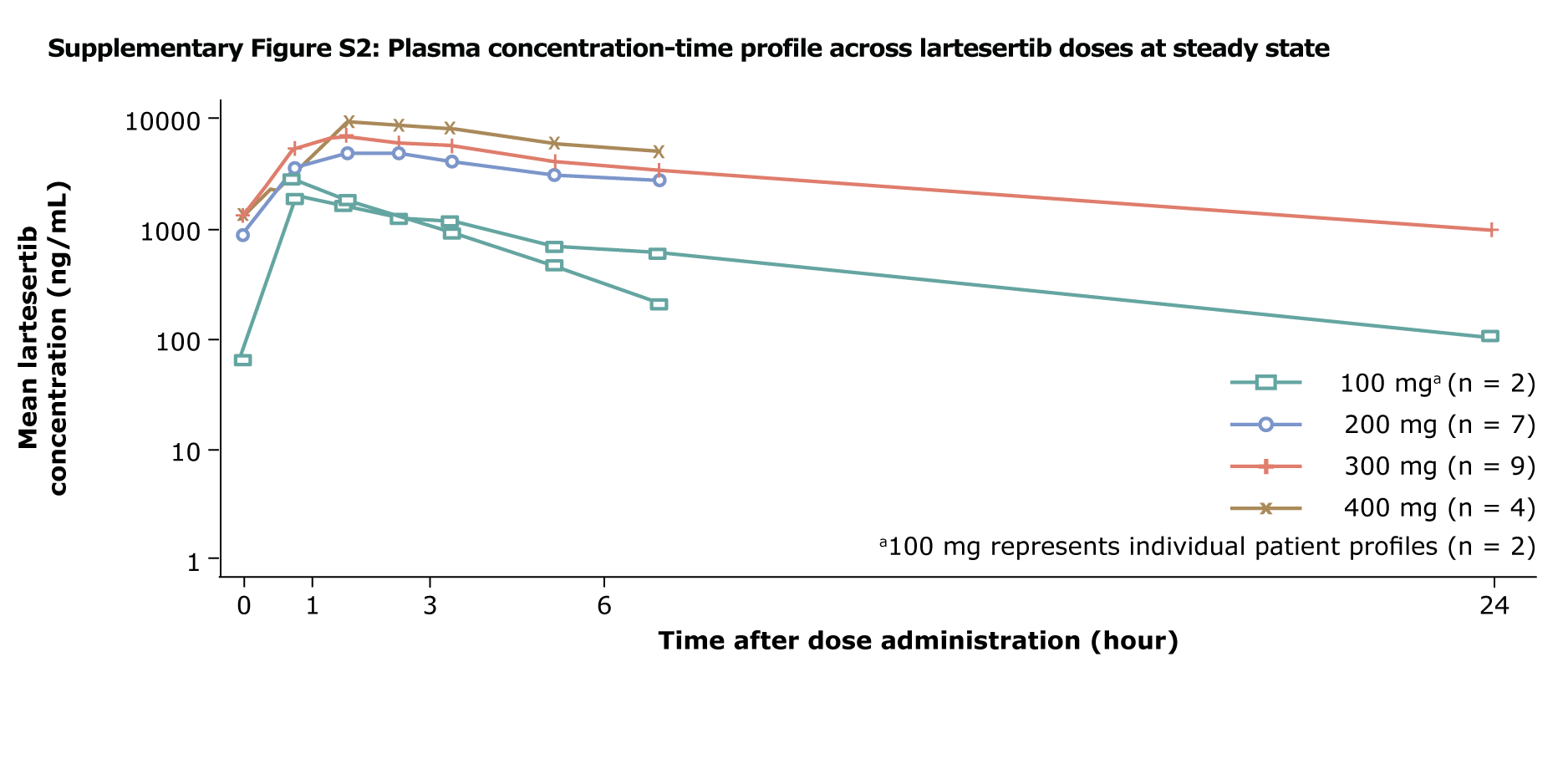

Supplement: Supplementary Figure S2 [file ccr-25-1648_supplementary_figure_s2_suppfs2.png]

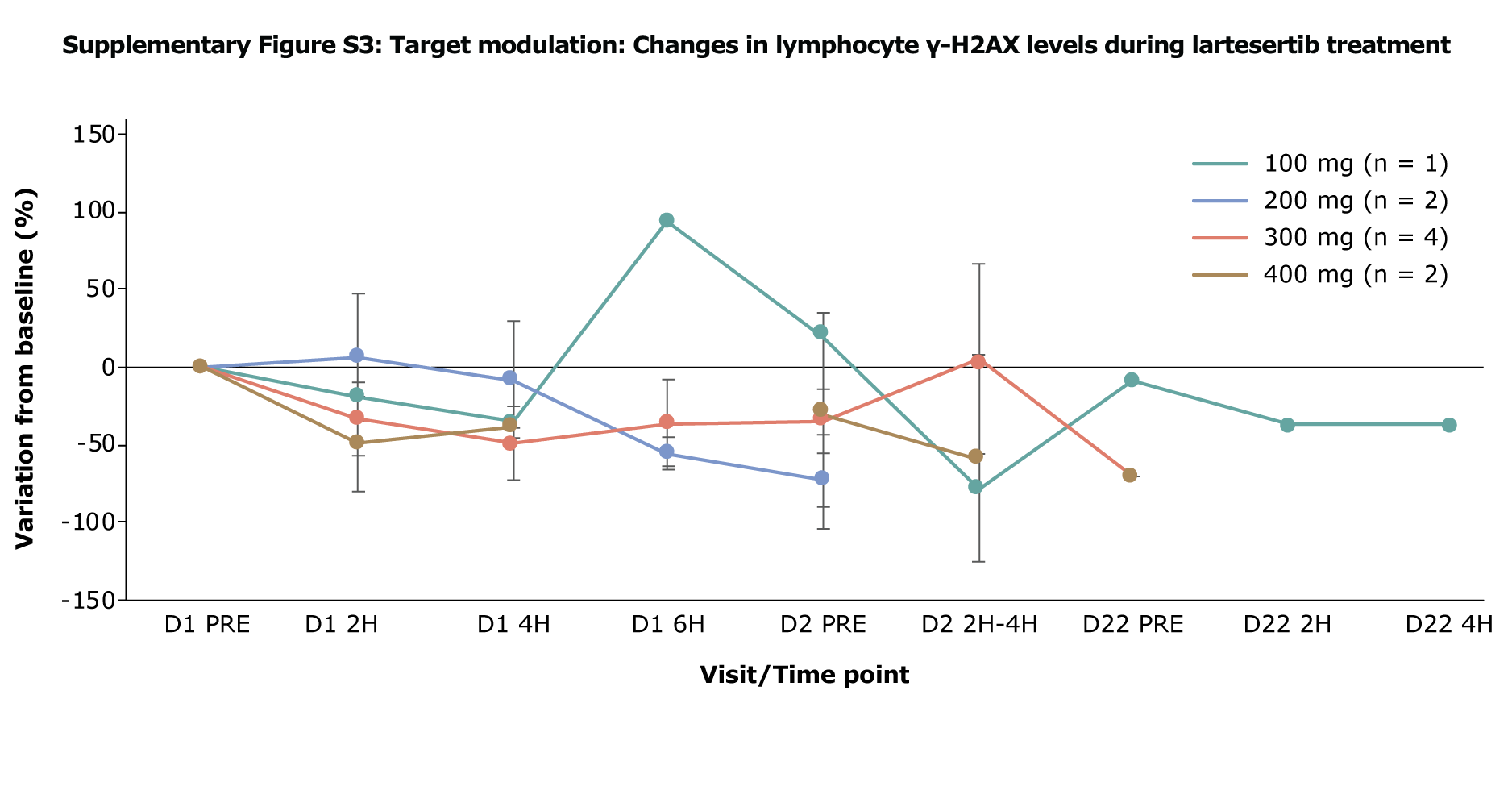

Supplement: Supplementary Figure S3 [file ccr-25-1648_supplementary_figure_s3_suppfs3.png]

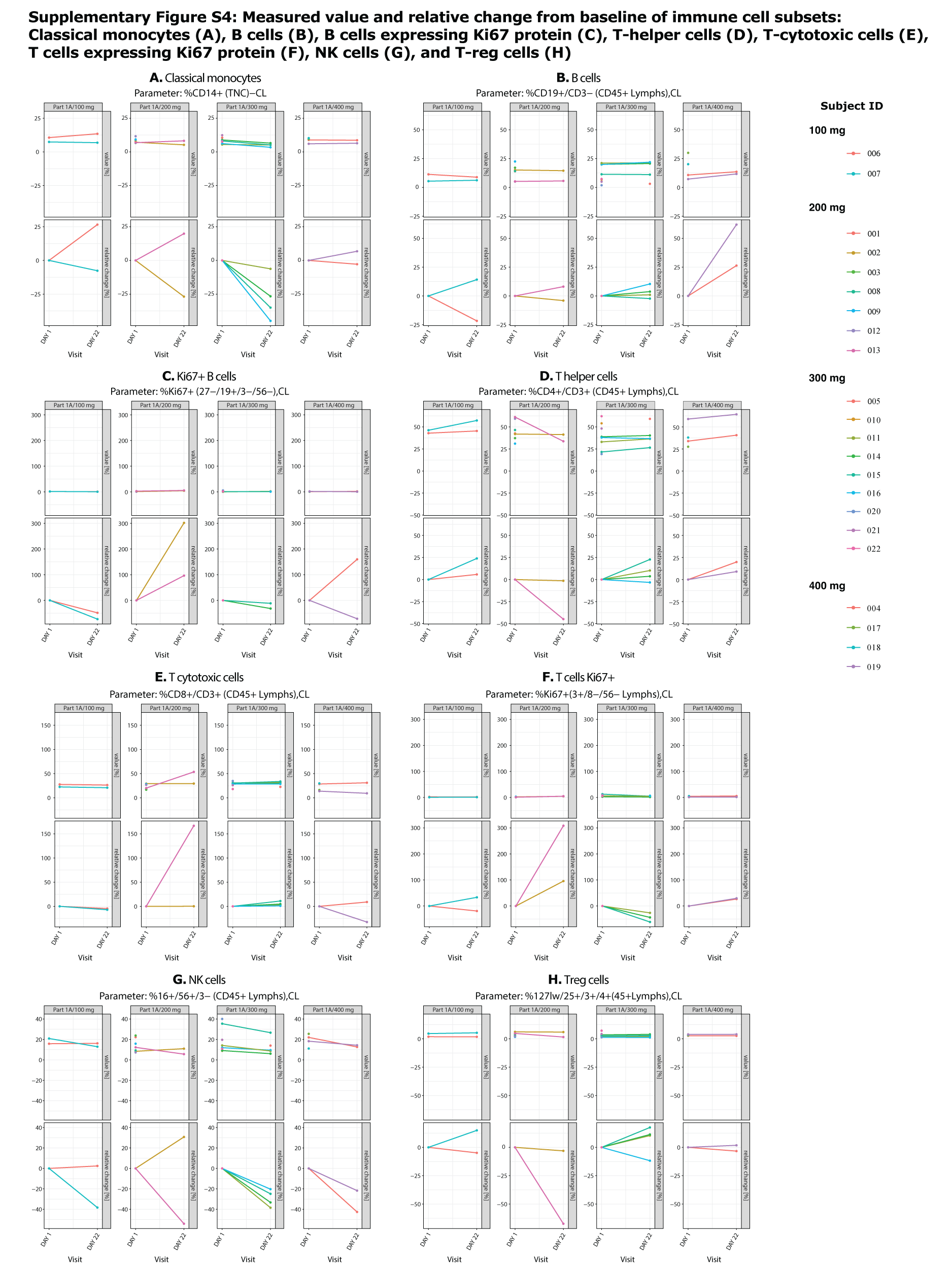

Supplement: Supplementary Figure S4 [file ccr-25-1648_supplementary_figure_s4_suppfs4.png]

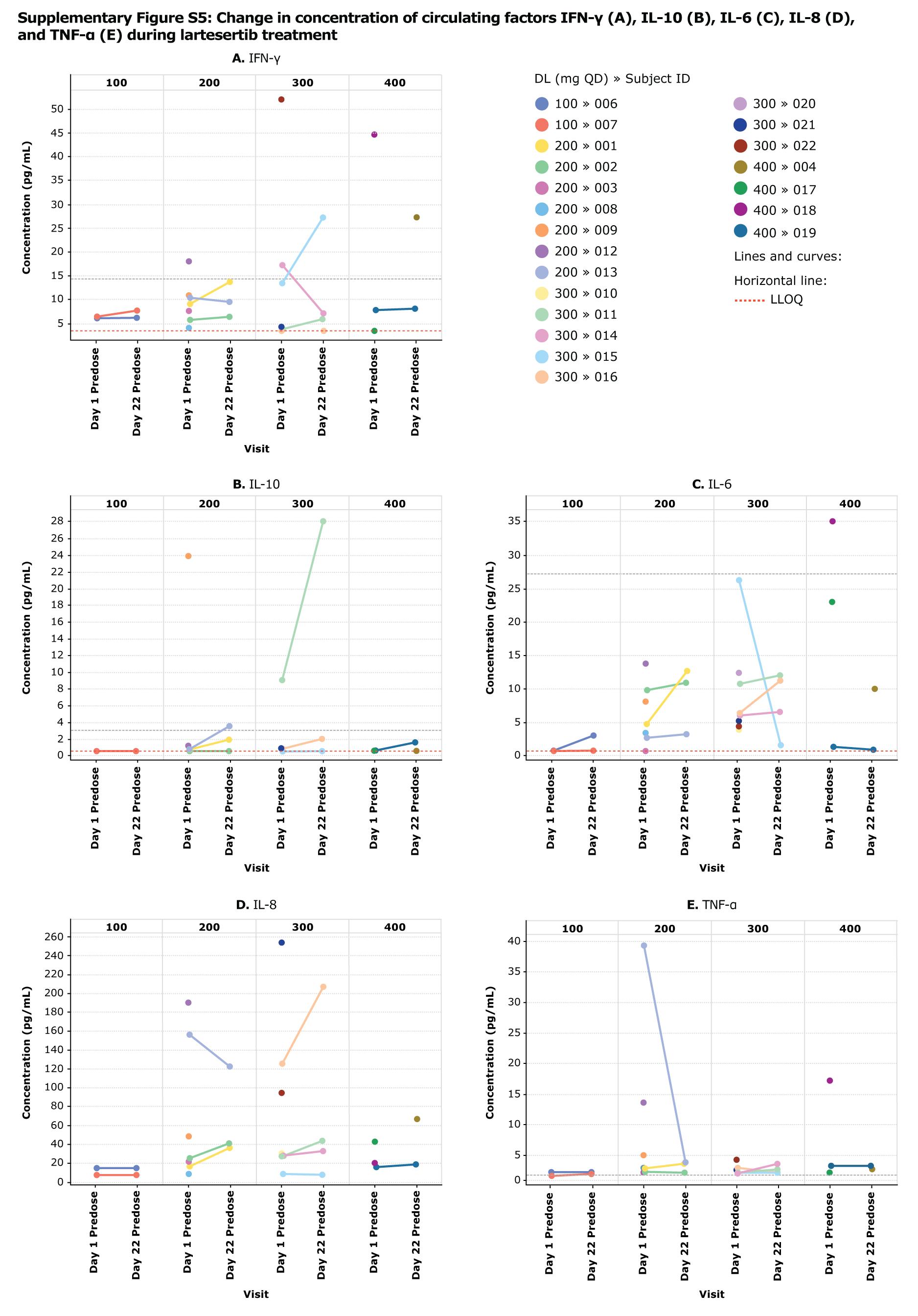

Supplement: Supplementary Figure S5 [file ccr-25-1648_supplementary_figure_s5_suppfs5.png]
